# The role of facility and patient mix factors on recovery of screening and diagnostic mammography volumes following the initial COVID‐19 pandemic wave

**DOI:** 10.1002/cam4.5793

**Published:** 2023-03-16

**Authors:** Sarah Lomahan, Garth H. Rauscher, Anne Marie Murphy

**Affiliations:** ^1^ Equal Hope Chicago Illinois USA; ^2^ Division of Epidemiology and Biostatistics University of Illinois at Chicago Chicago Illinois USA; ^3^ University of Illinois Cancer Center Chicago Illinois USA

**Keywords:** breast cancer, COVID‐19 pandemic, health disparities, mammography, quality improvement

## Abstract

**Introduction:**

The goal of this study was to understand the extent to which mammography facilities were able to recover monthly screening and diagnostic mammography volumes to their prepandemic levels and to determine what facility and patient mix factors were associated with recovery.

**Method:**

Facilities, located in and adjacent to Cook County, Illinois, were eligible. In all, 58 screening and 30 diagnostic mammogram facilities submitted mammogram volumes by month with a cross‐listing of patient ZIP codes by screening volumes. Monthly screening and diagnostic volumes for the 6‐month immediate postpandemic period (July–December 2020) and for the subsequent postpandemic period (January–June 2021) were compared with the same months in 2019. ZIP code distributions were used to define patient mix characteristics related to disadvantage.

**Results:**

Compared with the prepandemic period, Breast Imaging Centers of Excellence conducted roughly 50 fewer monthly screening mammograms (95% CI: −91, −9) but 50 more diagnostic mammograms (95% CI: 24, 82) on average in the immediate postpandemic period. Facilities serving a predominantly Black population conducted roughly 50 fewer monthly screens (95% CI: −93, −13) without any increase in monthly diagnostics.

**Conclusion:**

Highly accredited (and typically higher volume) facilities appeared to actively triage diagnostics, whereas lower resource facilities appeared to struggle to recover to prepandemic volumes without triage to diagnostics. The pandemic disproportionally impacted minority populations already affected by differential access to and utilization of high‐quality mammography. Potential explanations are discussed. Policies should be strengthened to facilitate triaging of services during times of stress to the healthcare system.

## Graphical abstract

We found that higher resource facilities appeared to actively triage diagnostics over screens, whereas lower resource facilities were generally not able to recover to prepandemic volumes and did not appear to triage diagnostics over screens. Additionally, we found that the pandemic disproportionally impacted disadvantaged and minority populations.

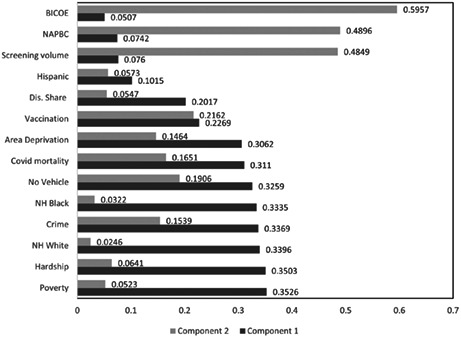

## 1 INTRODUCTION

The COVID‐19 pandemic exacerbated existing racial and ethnic health disparities with its disproportionate impact on communities of color through increased incidence, hospitalization, and mortality.^1^ During the first few months of the pandemic, COVID‐19 death rates were disproportionately higher for Black residents in Cook County, Illinois.^2^ In addition, COVID‐19 exacted an enormous toll on the overall healthcare system. With respect to preventive services both nationally and in Illinois, recommendations and executive orders were implemented to temporarily reduce nonessential health services to limit community spread and burden on the healthcare system.^3^, ^4^, ^5^ This resulted in a large temporary reduction in preventive services, including mammography.

Prior to the pandemic, relative to non‐Hispanic whites, ethnic minorities were less likely to undergo screening mammography, more likely to experience diagnostic mammography delays, and to receive healthcare services at less accredited facilities. Ethnic minorities were also more likely to attend hospitals serving a disproportionate share of uninsured and Medicaid patients known as Disproportionate Share Hospitals (DSH)^6^ and more likely to reside in areas where their community hospitals were facing closures.^2^, ^7^ Following the initial wave of the pandemic, many healthcare facilities struggled to recover their preventive services to prepandemic levels.^8^, ^9^, ^10^, ^11^, ^12^, ^13^, ^14^, ^15^, ^16^, ^17^, ^18^, ^19^, ^20^, ^21^, ^22^, ^23^, ^24^, ^25^, ^26^ In addition, hospitals were impacted by the pandemic due to increased demand, worker burnout, and safety guidelines.^27^ With respect to mammography, when facilities reopened many struggled to meet the increased demand due to missed screening and diagnostic mammograms.^9^, ^15^, ^20^

The study goal was to understand the extent to which mammography facilities were able to recover their monthly screening and diagnostic mammography volumes to prepandemic levels (i.e. levels in 2019); whether the difficulties facilities experienced in the immediate 6 months following the initial pandemic wave were sustained into the subsequent 6 months, and to determine what facility and patient mix factors contributed to the recovery of screening and diagnostic mammography volumes to prepandemic levels.

## 2 MATERIALS AND METHODS

Retrospective, aggregate data for these analyses were collected by Equal Care (a Chicagoland healthcare collaborative project of Equal Hope, formerly known as the Metropolitan Chicago Breast Cancer Task Force). Facilities located in and adjacent to Cook County, Illinois were eligible to participate if they performed screening mammograms throughout the period from January 1, 2019 to June 30, 2021; performed diagnostic mammograms throughout the same period; or both. Eligible facilities submitted monthly volumes of screening and diagnostic mammograms for the 30 months from January 2019 to June 2021. Facilities also generated a cross‐listing of the number of screening mammograms performed in 2019, 2020, and January–June 2021 by patient residential ZIP code. In all, 58 facilities that performed screening mammograms continuously throughout the study period, and 30 facilities that performed diagnostic mammograms continuously throughout the study period were included in these analyses.

The 30‐month study was grouped into two prepandemic and two postpandemic 6‐month time periods. The prepandemic reference period (baseline) was defined as the period from January 1, 2019 to December 31, 2019, divided into two, 6‐month periods. The immediate postpandemic period was defined as the first 6 months following the initial wave of the pandemic, from July 1, 2020 to December 31, 2020; and the subsequent postpandemic period was defined as the next 6 months following the pandemic period, from January 1, 2021 to June 30, 2021. The peripandemic period defined as the first 6 months of 2020 from January 1, 2020 to June 30, 2020 was not included in these analyses.

For each of the prepandemic and postpandemic 6‐month time periods, we calculated the average monthly screening and diagnostic volumes for each facility. We compared the average monthly volume (AMV) in the immediate postpandemic period (post) to the corresponding volumes for the same 6‐month period in 2019, before the pandemic (pre). We also compared AMV in the subsequent postpandemic period (post) to the corresponding volumes for the same 6‐month period in 2019, before the pandemic (pre). We calculated the change in AMV for each facility separately for screening and diagnostic volumes. For each, change was calculated in two ways by taking the difference in AMV for each facility:

$$\begin{array}{c} \text{Difference}=\sum_{i=1}^{N}\left(\text{AMVpost}-\text{AMVpre}\right) \end{array}$$

and by calculating the ratio of AMV (post)/AMV (pre):

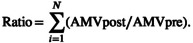




To examine whether a change in screening and diagnostic volumes was associated with facility characteristics, variables representing facility characteristics were defined. We defined whether a facility was accredited by the American College of Radiology as a Breast Imaging Center of Excellence (BICOE), accredited by the College of Surgeons National Accreditation Program for Breast Centers (NAPBC), and designated by the State of Illinois Medicaid Program as a DSH.^28^ BICOE accreditation requires that a facility have the full range of screening and diagnostic imaging available and can demonstrate a high level of quality in the implementation of breast imaging, including image‐guided biopsy procedures.^29^ NAPBC accreditation requires that a facility meet specific criteria regarding the range and quality of breast cancer diagnostic and treatment services offered.^30^ The monthly volume of screening mammograms performed prepandemic was also categorized into low (<250), medium (250–750), and high volumes (750 or more) to determine whether recovery to prepandemic volumes was better for higher or lower volume facilities.

Individual‐level data on patient sociodemographic characteristics were not available. As a proxy, we used the distribution of patient ZIP codes at facilities to define patient mix characteristics. For each facility, each ZIP code was weighted according to the proportion of patients screened at that facility residing in that ZIP code, with weights summing to 1.0 within each facility. From the American Community Survey, we pulled ZIP code level data on racial/ethnic composition, composite measures of disadvantage including hardship index, area deprivation index (ADI), percent poverty, and the percentage of adults with access to a vehicle. In addition, we pulled data on crime statistics, COVID‐19 mortality rates, and the percentage of adults with at least one COVID‐19 vaccination. The Hardship Index is a composite of six socioeconomic indicators (unemployment rate, age dependency, education, per capita income, crowded housing, and poverty).^31^ The ADI is a composite of 17 socioeconomic indicators, some of which overlap the Hardship Index.^32^ Separately for each facility and for each composite measure, we estimated a weighted average by summing ZIP code‐specific values of the composite measure across patient ZIP codes, with ZIP code‐specific values weighted according to the proportion of patients in each ZIP code. These patient mix factors were categorized roughly into thirds for analyses.

**FIGURE 1:**
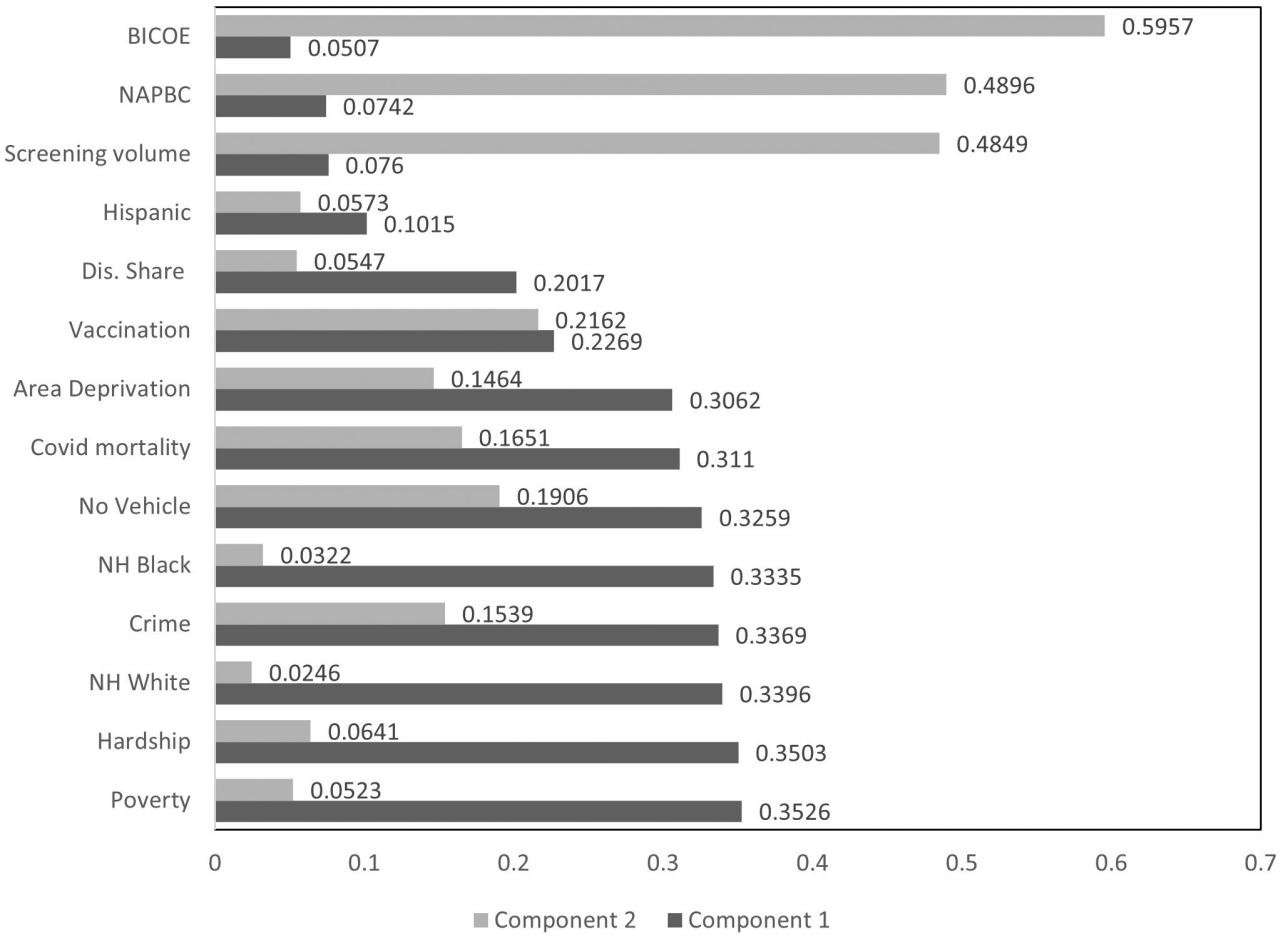
Graphical depiction of the extent to which each facility and patient mix factor loaded onto the first two components in principal components analysis. Lighter‐shaded bars represent loading onto the facility characteristics component and darker‐shaded bars represent loading onto the patient mix component.

Last, to reduce the dimensionality of our analysis for multivariable modeling, we conducted principal components analyses using screening volume, accreditation variables, DSH status, and facility patient mix variables. We extracted the first two components, which together accounted for 67% of the variance across the factors. Higher screening volume, BICOE, and NAPBC accreditation loaded strongly onto the first component, whereas DSH status, percent non‐Hispanic Black residents, crime, absence of a vehicle, hardship index, area deprivation index, poverty, and COVID‐19 mortality loaded strongly on the second component (Figure 1). The predicted values of these two components were standardized to have a mean of zero and a standard deviation of one and were modeled together in linear regression of screening volume recovery using robust standard errors.

## 3 RESULTS

### 3.1 Absolute screening volume recovery during the immediate postpandemic period

Table 1 presents the change in screening mammogram volumes comparing the first 6 months after the initial wave of the pandemic (immediate postpandemic period) to the monthly volumes for the same time period in the prior prepandemic year. Highest volume facilities (>750 monthly screening mammograms) were least able to recover from their prior screening volumes, conducting roughly 60 fewer mammograms per month on average than in 2019 (Difference = −59, 95% CI: −107, −10). Lowest volume facilities (<250 monthly screening mammograms) tended to return to prepandemic screening volumes (difference = 8, 95% CI: −34, 51). Overall, there was statistical evidence of a trend whereby as screening volume increased, the absolute recovery toward prepandemic levels decreased (p for trend = 0.04). Highly accredited BICOE and NAPBC facilities were also less likely to recover from their prior screening volumes compared with their nonaccredited counterparts, conducting roughly 50 and 43 fewer mammograms per month on average than in 2019. DSH hospitals were also less likely to recover from their prior screening volumes performing 55 fewer screening mammograms per month on average compared with the corresponding months in 2019 (Table 1).

**TABLE 1:** Extent to which facilities recovered monthly screening volumes during the immediate postpandemic period (July–December 2020) versus baseline (July–December 2019).

Characteristic	*N*	Range	Difference in screening volume			Ratio of screening volumes		
			Difference	(95% CI)	*p*	Ratio	(95% CI)	*p*
Screening volume	17	(<250)	8	(−34, 51)	0.04	1.06	(0.97, 1.15)	0.17
	27	(250–750)	−27	(−57, 2)		0.94	(0.88, 1.01)	
	10	(>750)	−59	(−107, −10)		0.97	(0.86, 1.08)	
BICOE	44	No	−14	(−40, 11)	0.15	0.99	(0.93, 1.05)	
	14	Yes	−50	(−91, −9)		0.95	(0.86, 1.04)	
NAPBC	44	No	−17	(−42, 9)	0.29	0.98	(0.92, 1.04)	0.99
	14	Yes	−43	(−85, −2)		0.98	(0.89, 1.07)	
DSH	44	No	−15	(−39, 10)	0.12	1.00	(0.94, 1.05)	
	14	Yes	−55	(−99, −10)		0.92	(0.82, 1.02)	
% NH White	20	(8–30)	−41	(−78, −3)	0.05	0.90	(0.82, 0.98)	0.00
	19	(32–59)	−43	(−78, −7)		0.96	(0.89, 1.04)	
	19	(60–81)	11	(−26, 47)		1.07	(0.99, 1.15)	
% NH Black	20	(1–6)	9	(−26, 43)	0.02	1.08	(1.01, 1.15)	
	19	(7–24)	−36	(−71, −1)		0.92	(0.85, 1.00)	
	19	(26–78)	−53	(−93, −13)		0.92	(0.83, 1.00)	
% Hispanic	20	(9–15)	−11	(−49, 27)	0.40	0.99	(0.91, 1.07)	0.27
	19	(16–26)	−27	(−65, 11)		1.02	(0.94, 1.11)	
	19	(26–64)	−34	(−72, 4)		0.92	(0.84, 1.01)	
% Poverty	20	(4–8)	5	(−31, 41)	0.07	1.07	(0.99, 1.14)	
	19	(8–15)	−38	(−74, −2)		0.96	(0.88, 1.03)	
	19	(17–28)	−43	(−82, −3)		0.90	(0.81, 0.98)	
Area deprivation	20	(25–36)	3	(−33, 39)	0.08	1.06	(0.99, 1.14)	0.00
	19	(37–50)	−37	(−72, −1)		0.96	(0.88, 1.03)	
	19	(50–71)	−43	(−84, −2)		0.90	(0.81, 0.98)	
Hardship index	20	(17–37)	−3	(−40, 33)	0.17	1.05	(0.98, 1.13)	
	19	(38–59)	−32	(−67, 4)		0.98	(0.90, 1.05)	
	19	(60–81)	−40	(−82, 1)		0.89	(0.80, 0.97)	
Crime rate	20	(2–115)	18	(−15, 51)	0.02	1.07	(1.00, 1.15)	0.00
	19	(177–2209)	−58	(−94, −22)		0.93	(0.85, 1.01)	
	19	(2326–4592)	−40	(−76, −4)		0.92	(0.84, 1.00)	
% No vehicle	20	(3–5)	12	(−21, 46)	0.05	1.03	(0.95, 1.11)	
	19	(6–19)	−55	(−92, −19)		0.97	(0.89, 1.05)	
	19	(20–32)	−36	(−73, 1)		0.92	(0.84, 1.01)	
Covid mortality	20	(0–9)	12	(−22, 46)	0.04	1.03	(0.96, 1.11)	0.01
	19	(10–135)	−52	(−89, −16)		1.01	(0.93, 1.09)	
	19	(137–191)	−39	(−76, −2)		0.89	(0.80, 0.97)	
Vaccination	20	(55–67)	−44	(−84, −4)		0.93	(0.84, 1.02)	
	19	(67–73)	−15	(−51, 21)		0.97	(0.89, 1.05)	
	19	(73–77)	−17	(−55, 21)		1.03	(0.95, 1.11)	

*Note*: *p* values >0.20 are suppressed.

With respect to patient mix, facilities whose patients resided in ZIP codes associated with greater disadvantage (a lower proportion of non‐Hispanic white residents, a higher proportion of non‐Hispanic Black residents, greater hardship, area deprivation, and poverty indices) were less likely to recover from their prior screening volumes compared with facilities with a more advantaged patient mix. Facilities whose patients resided in ZIP codes with greater crime, higher COVID‐19 mortality rates, and lower COVID‐19 vaccination rates appeared somewhat less likely to recover from their prior screening volumes (Table 1).

### 3.2 Percent of relative screening volume recovery during the immediate postpandemic period

When changes in screening volumes were presented as ratios, patterns of screening volume recovery were affected by the baseline (prepandemic) volume of each facility. For example, whereas higher volume facilities had a lower absolute screening volume recovery compared with lower volume facilities, when expressed as a ratio, the decrease in volume postpandemic of approximately 60 screening mammograms was only a 3% reduction, and the trend evident for absolute change was less apparent when expressed as a ratio. From a public health perspective, however, it is the absolute change that matters most.

### 3.3 Absolute diagnostic volume recovery during the immediate postpandemic period

Table 2 presents the change in monthly diagnostic mammogram volumes comparing the first 6 months after the initial wave of the pandemic (immediate postpandemic period) compared with monthly diagnostic volumes for the same time period in the prior year. Higher volume and more highly accredited BICOE and NAPBC facilities appeared to prioritize diagnostic mammograms, conducting roughly 50 more diagnostic mammograms per month in the immediate postpandemic period compared with baseline, whereas non‐BICOE/NAPBC facilities had diagnostic volumes equivalent to prepandemic levels. Both DSH facilities and non‐DSH facilities recovered their monthly diagnostic volumes in the immediate postpandemic period compared with baseline (Table 2).

**TABLE 2:** Extent to which facilities recovered monthly diagnostic volumes during the immediate postpandemic period (July–December 2020) versus baseline (July–December 2019).

Characteristic	*N*	Range	Difference in diagnostic volume			Ratio of diagnostic volumes		
			Difference	(95% CI)	*p*	Ratio	(95% CI)	*p*
BICOE	19	No	−8	(−37, 21)	0.00	0.94	(0.86, 1.02)	<0.001
	11	Yes	53	(24, 82)		1.15	(1.07, 1.24)	
NAPBC	20	No	−3	(−31, 26)	0.01	0.99	(0.90, 1.08)	0.04
	10	Yes	52	(21, 83)		1.12	(1.02, 1.22)	
DSH	18	No	29	(1, 56)	0.39	1.06	(0.97, 1.14)	
	12	Yes	6	(−40, 51)		1.02	(0.89, 1.16)	
% NH White	13	(8–30)	20	(−26, 66)	0.56	1.01	(0.87, 1.15)	0.34
	9	(32–59)	12	(−27, 50)		1.03	(0.92, 1.15)	
	8	(60–70)	37	(−4, 77)		1.10	(0.98, 1.22)	
% NH Black	7	(3–6)	45	(3, 87)	0.12	1.16	(1.05, 1.28)	0.01
	11	(7–24)	23	(−14, 61)		1.03	(0.92, 1.13)	
	12	(26–75)	−1	(−43, 41)		0.96	(0.84, 1.07)	
% Hispanic	7	(9–15)	28	(−15, 72)	0.92	1.06	(0.93, 1.19)	0.93
	13	(16–26)	15	(−24, 55)		1.04	(0.92, 1.15)	
	10	(26–59)	25	(−18, 69)		1.05	(0.92, 1.18)	
% Poverty	7	(5–7)	44	(1, 86)	0.19	1.13	(1.00, 1.25)	0.06
	11	(8–15)	19	(−16, 53)		1.04	(0.94, 1.14)	
	12	(17–28)	2	(−47, 50)		0.95	(0.81, 1.09)	
Area deprivation	10	(27–36)	56	(19, 92)	0.26	1.14	(1.03, 1.25)	0.18
	9	(39–50)	−10	(−45, 24)		0.98	(0.87, 1.08)	
	11	(50–68)	27	(−15, 68)		1.03	(0.90, 1.16)	
Hardship index	8	(20–36)	51	(13, 90)		1.12	(1.01, 1.24)	0.16
	11	(39–59)	−1	(−35, 34)		1.01	(0.91, 1.12)	
	11	(60–81)	22	(−25, 69)		1.00	(0.86, 1.15)	
Crime rate	6	(5–44)	26	(−17, 69)	0.49	1.10	(0.97, 1.22)	0.15
	10	(269–2209)	30	(−5, 65)		1.06	(0.96, 1.17)	
	14	(2326–4592)	2	(−48, 51)		0.95	(0.81, 1.10)	
% No vehicle	6	(3–5)	26	(−17, 70)		1.10	(0.97, 1.22)	
	11	(7–19)	29	(−8, 66)		1.05	(0.95, 1.16)	
	13	(20–32)	8	(−38, 54)		0.98	(0.85, 1.12)	
Covid mortality	6	(0–2)	26	(−17, 70)	0.88	1.10	(0.97, 1.23)	0.35
	12	(10–135)	20	(−14, 55)		1.04	(0.94, 1.14)	
	12	(137–191)	22	(−33, 78)		1.00	(0.84, 1.17)	
Vaccination	10	(57–67)	15	(−24, 54)		0.99	(0.87, 1.10)	0.12
	11	(68–73)	2	(−39, 44)		1.04	(0.92, 1.17)	
	9	(73–77)	48	(9, 87)		1.12	(1.00, 1.23)	

*Note*: *p* values >0.20 are suppressed.

Facilities whose patients resided in ZIP codes associated with greater disadvantage were somewhat less likely to recover to their prior diagnostic volumes compared to those with a more advantaged patient mix, though statistical evidence was more modest (Table 2).

### 3.4 Percent or relative diagnostic volume recovery during the immediate postpandemic period

When changes in diagnostic volumes were presented as ratios, patterns of recovery were qualitatively similar to results on the absolute scale. For example, at BICOE facilities, monthly diagnostic volumes were 15% greater in the immediate postpandemic period compared with baseline (ratio = 1.15, 95% CI: 1.07, 1.24), whereas for non‐BICOE facilities, they were 6% lower (ratio = 0.94, 95% CI: 0.86, 1.02) (p for difference in these ratios = <0.001). Similar results were observed for NAPBC versus non‐NAPBC facilities (Table 2).

### 3.5 Absolute and relative screening volume recovery during the subsequent postpandemic period

Table 3 presents the change in screening mammogram volumes comparing the subsequent 6‐month postpandemic period (January–June 2021) to monthly volumes for the same time period in 2019. Higher volume facilities continued to be least likely to recover from their prior screening volumes, conducting roughly 89 fewer mammograms per month on average than they were in 2019. Lowest volume facilities, on the other hand, tended to return to prepandemic screening volumes and there was statistical evidence of a trend, whereby as screening volume increased, the absolute recovery toward prepandemic levels decreased (*p* for trend = 0.03).

**TABLE 3:** Extent to which facilities recovered monthly screening volumes for the subsequent postpandemic period (January–June 2021) versus baseline (January–June 2019).

Characteristic	*N*	Range	Difference in screening volume			Ratio of screening volumes		
			Difference	(95% CI)	*p*	Ratio	(95% CI)	*p*
Screening volume	12	(<250)	2	(−57, 61)	0.03	1.00	(0.90, 1.11)	0.45
	27	(250–750)	−27	(−61, 7)		0.94	(0.88, 1.01)	
	10	(>750)	−89	(−145, −33)		0.94	(0.84, 1.05)	
BICOE	6	No	−20	(−51, 10)	0.18	0.97	(0.91, 1.03)	
	13	Yes	−60	(−108, −12)		0.95	(0.86, 1.04)	
NAPBC	35	No	−19	(−50, 12)	0.12	0.96	(0.91, 1.02)	0.93
	14	Yes	−64	(−112, −16)		0.96	(0.87, 1.05)	
DSH	38	No	−31	(−61, −2)		0.97	(0.92, 1.02)	
	11	Yes	−35	(−93, 23)		0.94	(0.83, 1.04)	
% NH White	15	(8–30)	−49	(−98, 0)	0.48	0.87	(0.79, 0.95)	0.05
	17	(32–59)	−25	(−69, 18)		1.01	(0.94, 1.08)	
	17	(60–81)	−24	(−71, 22)		0.99	(0.91, 1.07)	
% NH Black	19	(1–6)	−14	(−57, 29)		1.00	(0.93, 1.08)	
	17	(7–24)	−52	(−96, −7)		0.95	(0.87, 1.03)	
	13	(26–71)	−32	(−83, 19)		0.92	(0.83, 1.01)	
% Hispanic	16	(9–15)	−15	(−61, 31)	0.41	1.00	(0.92, 1.08)	0.13
	17	(16–26)	−39	(−85, 7)		0.99	(0.90, 1.07)	
	16	(26–64)	−42	(−88, 4)		0.91	(0.83, 0.99)	
% Poverty	18	(4–8)	−12	(−57, 32)		1.00	(0.92, 1.08)	
	17	(8–15)	−41	(−84, 2)		1.00	(0.92, 1.07)	
	14	(17–25)	−46	(−96, 5)		0.87	(0.78, 0.95)	
Area deprivation	18	(25–36)	−50	(−95, −6)	0.66	0.98	(0.90, 1.06)	0.19
	18	(37–50)	−8	(−53, 36)		1.00	(0.93, 1.08)	
	13	(50–68)	−38	(−87, 10)		0.90	(0.81, 0.98)	
Hardship index	18	(17–37)	−41	(−85, 2)		0.99	(0.91, 1.06)	
	18	(38–59)	−1	(−43, 41)		1.02	(0.95, 1.09)	
	13	(60–81)	−62	(−112, −13)		0.85	(0.77, 0.93)	
Crime rate	19	(2–115)	−8	(−50, 35)	0.28	0.99	(0.92, 1.07)	0.12
	15	(177–2209)	−51	(−95, −7)		0.98	(0.90, 1.06)	
	15	(2326–3984)	−41	(−91, 10)		0.90	(0.81, 0.99)	
% No vehicle	19	(3–5)	−3	(−45, 38)	0.16	1.01	(0.94, 1.09)	
	15	(6–19)	−56	(−100, −11)		0.96	(0.88, 1.04)	
	15	(20–29)	−45	(−95, 5)		0.89	(0.80, 0.98)	
Covid mortality	19	(0–9)	−3	(−45, 38)	0.10	1.01	(0.94, 1.08)	0.01
	15	(10–135)	−48	(−94, −1)		1.00	(0.92, 1.07)	
	15	(137–185)	−54	(−102, −6)		0.86	(0.78, 0.94)	
Vaccination	14	(57–67)	−17	(−64, 31)		0.97	(0.89, 1.06)	
	18	(67–73)	−28	(−73, 16)		0.96	(0.88, 1.04)	
	17	(73–77)	−50	(−96, −4)		0.96	(0.88, 1.04)	

*Note*: *p* values of >0.20 are suppressed.

### 3.6 Absolute and relative diagnostic volume recovery during the subsequent postpandemic period

Table 4 presents the change in diagnostic mammogram volumes comparing the subsequent postpandemic period to the same time period in 2019. Again, more highly accredited, higher volume BICOE and NAPBC facilities appeared to continue to prioritize diagnostic mammograms, conducting roughly 50 more diagnostic mammograms per month in the postpandemic period compared with baseline, whereas non‐BICOE and non‐NAPBC accredited facilities had diagnostic volumes equivalent to prepandemic levels. Expressed as a ratio, BICOE and NAPBC facilities performed 16% and 13% more diagnostic mammograms in the subsequent postpandemic period compared with baseline, whereas non‐BICOE performed 3% less and non‐NAPBC had no change.

**TABLE 4:** Extent to which facilities recovered monthly diagnostic volumes for the subsequent postpandemic period (January–June 2021) versus baseline (January–June 2019).

Characteristic	*N*	Range	Difference in diagnostic volume			Ratio of diagnostic volumes		
			Difference	(95% CI)	*p*	Ratio	(95% CI)	*p*
BICOE	19	No	−2	(−46, 41)	0.10	0.97	(0.85, 1.08)	0.02
	11	Yes	50	(5, 96)		1.16	(1.04, 1.28)	
NAPBC	20	No	−2	(−46, 42)	0.11	1.00	(0.88, 1.12)	0.15
	10	Yes	50	(4, 95)		1.13	(1.00, 1.25)	
DSH	18	No	31	(−8, 69)		1.09	(0.98, 1.19)	
	12	Yes	4	(−59, 66)		0.99	(0.82, 1.16)	
% NH White	13	(8–30)	34	(−29, 97)	0.85	1.07	(0.90, 1.24)	0.76
	9	(32–59)	−2	(−57, 54)		1.01	(0.86, 1.16)	
	8	(60–70)	39	(−16, 94)		1.10	(0.95, 1.25)	
% NH Black	7	(3–6)	49	(−10, 108)		1.16	(1.00, 1.32)	0.15
	11	(7–24)	13	(−43, 68)		1.02	(0.87, 1.17)	
	12	(26–75)	9	(−50, 67)		1.00	(0.85, 1.16)	
% Hispanic	7	(9–15)	31	(−27, 89)	0.75	1.09	(0.93, 1.25)	0.91
	13	(16–26)	−1	(−53, 51)		1.01	(0.87, 1.16)	
	10	(26–59)	48	(−15, 110)		1.08	(0.91, 1.26)	
% Poverty	7	(5–7)	45	(−14, 104)		1.12	(0.96, 1.27)	0.17
	11	(8–15)	16	(−34, 66)		1.07	(0.94, 1.21)	
	12	(17–28)	6	(−62, 74)		0.95	(0.77, 1.13)	
Area deprivation	10	(27–36)	37	(−17, 91)	0.93	1.12	(0.98, 1.27)	0.77
	9	(39–50)	−8	(−62, 46)		0.96	(0.81, 1.10)	
	11	(50–68)	45	(−17, 107)		1.10	(0.94, 1.27)	
Hardship index	8	(20–36)	28	(−29, 84)		1.06	(0.93, 1.23)	
	11	(39–59)	15	(−39, 69)		1.07	(0.92, 1.22)	
	11	(60–81)	29	(−40, 98)		1.01	(0.82, 1.19)	
Crime rate	6	(5–44)	30	(−30, 90)	0.61	1.11	(0.95, 1.26)	0.21
	10	(269–2209)	27	(−24, 78)		1.08	(0.95, 1.22)	
	14	(2326–4592)	6	(−63, 75)		0.95	(0.77, 1.13)	
% No vehicle	6	(3–5)	30	(−30, 90)		1.11	(0.95, 1.27)	
	11	(7–19)	23	(−30, 77)		1.07	(0.92, 1.21)	
	13	(20–32)	14	(−50, 78)		0.99	(0.82, 1.16)	
Covid mortality	6	(0–2)	30	(−29, 90)	0.99	1.11	(0.94, 1.27)	0.43
	12	(10–135)	13	(−36, 62)		1.05	(0.92, 1.18)	
	12	(137–191)	36	(−40, 111)		1.01	(0.80, 1.21)	
Vaccination	10	(57–67)	39	(−17, 94)		1.07	(0.92, 1.22)	
	11	(68–73)	−6	(−69, 57)		0.99	(0.82, 1.16)	
	9	(73–77)	30	(−25, 85)		1.10	(0.95, 1.25)	

*Note*: *p* values >0.20 are suppressed.

### 3.7 Multivariable models of screening and diagnostic volume recovery in the immediate 6‐month postpandemic period

A one standard deviation increase in the patient disadvantage score was associated with a deficit of 22 monthly screening mammograms (95% CI: −42, 2), and a deficit of 25 monthly diagnostic mammograms (95% CI: ‐48, 2). In contrast, a one standard deviation increase in the volume and accreditation score was associated with a deficit of 25 screening mammograms per month, but was also associated with an increase of 34 diagnostic mammograms per month. (Table 5).

**TABLE 5:** Multivariable models of the extent to which facilities recovered their monthly screening volumes for the immediate postpandemic period (July–December 2020) versus baseline (July–December 2019).

	Difference^a^	(95% CI)	*p* value
Immediate postpandemic period			
Screening			
Component 1 (Greater Disadvantage)	−22	(−42, −2)	0.03
Component 2 (Greater Volume and Accreditation)	−25	(−48, −2)	0.04
Diagnostic			
Component 1 (Greater Disadvantage)	−14	(−41, 14)	
Component 2 (Greater Volume and Accreditation)	34	(8, 59)	0.01
Subsequent postpandemic period			
Screening			
Component 1 (Greater Disadvantage)	−8	(−34, 18)	
Component 2 (Greater Volume and Accreditation)	−34	(−70, 2)	0.07
Diagnostic			
Component 1 (Greater Disadvantage)	−7	(−55, 42)	
Component 2 (Greater Volume and Accreditation)	24	(−18, 65)	

*Note*: *p* values of 0 > 0.20 are suppressed.

^a^ Absolute change in monthly volume (post vs. prepandemic) for a one standard deviation change in each principal component score.

### 3.8 Multivariable models of screening and diagnostic volume recovery in the subsequent 6‐month postpandemic period

The patient disadvantage score was not substantially associated with either a deficit or an excess of screening or diagnostic mammograms for the subsequent postpandemic compared with baseline. In contrast, a one standard deviation increase in the volume and accreditation score was marginally associated with a deficit of 34 screening mammograms per month with a corresponding increase of 24 monthly diagnostic mammograms (Table 5).

## 4 DISCUSSION

Prior studies have observed postpandemic reductions in screening mammography volume experienced within a single healthcare system,^9^, ^18^, ^19^, ^20^, ^21^, ^22^ within highly accredited facilities^17^ or across a large mammography registry.^15^ In this study of diverse healthcare entry points, including large multihospital healthcare systems, academic hospitals, community hospitals, DSH hospitals, and accredited and nonaccredited facilities, we attempted to create a comprehensive picture of how the postpandemic recovery to prepandemic mammography levels varied based on facility characteristics and patient mix across a large racially and ethnically diverse metropolitan area.

There appeared to be two separate processes operating that affected whether facilities recovered their screening volumes to prepandemic levels. First, larger volume and more highly accredited facilities appeared to be triaging diagnostics in favor of screening mammograms, which could explain their reduced screening volume relative to the period prior to the pandemic. A previous survey of facilities found that the vast majority of facilities self‐reported prioritizing diagnostic imaging over screening^33^ and that diagnostic volumes tended to reach or exceed prepandemic levels, overcompensating for the loss during temporary closures.^15^, ^24^, ^25^ However, self‐reported data can suffer from social desirability bias and the extent to which the facilities were triaging is unclear. In the current study, we observed evidence for such triaging at the larger volume and accredited sites but not at DSH. Performing diagnostic mammograms requires a radiologist to be onsite and many non‐academic and DSH contract with external radiology groups rather than employing radiologists directly. This may mean that they have less flexibility to prioritize diagnostic mammograms and are less likely to be in hospital leadership regarding discussions of triaging practices.^34^ Radiology groups also suffered very significant revenue decreases during the pandemic, and this may present another set of challenges.^35^

Facilities with a disadvantaged patient mix were less likely to recover their screening volume to prepandemic levels, despite the lack of any apparent triaging toward diagnostic imaging. Facilities with a disadvantaged patient mix may also have fewer resources due to the nature of their patient population which tends to be uninsured and underinsured. A nationwide survey from March 25 to April 8, 2020 found that 38% of respondents said COVID‐19 adversely affected their ability to afford medical care and led many to forgo care.^36^ Given the patient population served by DSH facilities, this likely affected them disproportionately. Lack of sufficient funding streams could result in difficulty maintaining operations and retaining staff, and the pandemic may have exacerbated these challenges.

Initially, all available staff was utilized to triage and treat COVID‐19 patients, frequently resulting in burnout and “compassion fatigue,” which further exacerbated staff shortages.^37^, ^38^ DSH providers may themselves have experienced a greater personal burden due to the pandemic compared with staff at better‐funded facilities, potentially resulting in greater burnout and quit rates. All of the above could help to explain the inability of DSH facilities to recover to their prepandemic screening volumes and the absence of triaging to diagnostic mammography.

Black and Brown communities in Chicago have experienced higher rates of COVID‐19 infection and mortality, lower rates of vaccination coverage, and may have a greater perception of risk of contracting COVID‐19 if traveling for preventive care.^39^, ^40^ In these communities, a higher percentage of households lack access to a vehicle thus requiring public transportation. Public transportation ridership in Chicago dropped by three‐fourths during the pandemic, causing a reduction in services, further exacerbating this structural barrier to transportation to healthcare.^41^, ^42^ Other volunteer rideshare programs for low‐income patients were also suspended due to the pandemic for an extended period of time.^43^ A greater percentage of those losing employment were from disadvantaged areas, leading to greater residential mobility which is also associated with reduced preventive care.^44^

This study has limitations. Despite a large number of patients, our sample size was limited to 58 screening and 30 diagnostic facilities, which limited our ability to obtain precise estimates for changes in monthly mammogram volumes. In addition, while studies from single institutions or healthcare organizations can access individual patient data, our multi‐institutional study lacked individual‐level data on patient characteristics. We used the distributions of patient ZIP codes to define proxy measures for the patient mix. This study also has several strengths. We utilized a large and diverse sample of mammography facilities across Chicago, Cook County, and adjacent to Cook County, including large healthcare systems, academic hospitals, community hospitals, and DSH to create a more granular and population perspective regarding the impact of the pandemic on screening and diagnostic mammography utilization.

The pandemic's effects on screening and diagnostic mammography have the potential to exacerbate existing racial and ethnic breast cancer inequities, including a shift toward later stages of diagnoses.^44^, ^45^ COVID‐19 will not be the only pandemic to affect preventive services, and there needs to be greater financial support for facilities with fewer resources so that disadvantaged patients are not disproportionally affected. Policies should be strengthened to facilitate optimal triaging of breast and other healthcare services during times of stress to the healthcare system.^46^

## AUTHOR CONTRIBUTIONS

**Sarah Lomahan:** Data curation (equal); formal analysis (equal); writing – original draft (equal); writing – review and editing (equal). **Garth H Rauscher:** Conceptualization (equal); formal analysis (equal); writing – original draft (equal); writing – review and editing (equal). **Anne Marie Murphy:** Conceptualization (equal); data curation (equal); formal analysis (equal); writing – original draft (equal); writing – review and editing (equal).

## FUNDING INFORMATION

This work was supported by grants from the Polk Bros Foundation and the Healthy Communities Foundation as well as by the Population Health Analytics, Metrics and Evaluation Center (PHAME) at the University of Illinois at Chicago. The authors declare that they had full access to all data in this study, and the authors take complete responsibility for the integrity of the data and the accuracy of the analysis.

## ETHICS STATEMENT

The study was determined to be exempt by the Rush University Institutional Review Board prior to commencing this study.

## Data Availability

The data that support the findings of this study are available from the corresponding author upon reasonable request.
